# Sweet taste receptor inhibitors: Potential treatment for equine insulin dysregulation

**DOI:** 10.1371/journal.pone.0200070

**Published:** 2018-06-29

**Authors:** Melody Anne de Laat, Murad Hasan Kheder, Christopher Charles Pollitt, Martin Nicholas Sillence

**Affiliations:** 1 School of Earth, Environmental and Biological Sciences, Queensland University of Technology, Brisbane, Queensland, Australia; 2 School of Veterinary Science, The University of Queensland, Gatton, Queensland, Australia; Leibniz-Institute for Food Systems Biology at the TU Munich, GERMANY

## Abstract

Hyperinsulinemia is a major risk factor for equine laminitis, a debilitating and painful foot condition. Sweet taste receptor (T1R2/3) inhibitors have been used to reduce the insulin and glucose responses to oral carbohydrates in other species. However, their effect in horses has not been investigated. It would be useful to be able to attenuate the large post-prandial insulin response that typically occurs when a carbohydrate-rich meal is fed to insulin-dysregulated horses. Here we have determined the efficacy of two T1R2/3 inhibitors, lactisole and *Gymnema sylvestre*, for reducing glucose uptake by the equine small intestine *in vitro;* and post-prandial insulin secretion in ponies *in vivo*, following a carbohydrate-based meal. We used gas chromatography-mass spectrometry to measure 2-deoxyglucose uptake by explants of small intestine, in the presence and absence of the T1R2/3 inhibitors. Lactisole and *G sylvestre* reduced 2-deoxyglucose uptake by the intestinal explants by 63% (*P* = 0.032) and 73% (*P* = 0.047), respectively, compared to control samples. The study *in vivo* investigated the effect of the inhibitors on the blood glucose and serum insulin responses to a meal containing D-glucose. Three doses of each inhibitor were tested using a Latin square design, and each dose was compared to a meal with no inhibitor added. Lactisole had no effect on glucose and insulin concentrations, whereas *G sylvestre* was partially effective at reducing post-prandial blood glucose (by ~10%) and serum insulin concentrations (~25%) in seven ponies, with a most effective dose of 10 mg/kg bodyweight. These data provide preliminary support that T1R2/3 inhibitors may be a useful therapeutic strategy for the management of equine insulin dysregulation and the prevention of laminitis. However, further optimisation of the dose and delivery method for these compounds is required, as well as a direct investigation of their activity on the equine sweet taste receptor.

## Introduction

Laminitis is a painful foot disease of ungulates in which the epidermal lamellae that connect the distal phalanx and the inner hoof wall fail, resulting in distal phalanx dislocation and often, euthanasia of the animal [1]. It is well-established that hyperinsulinemia is a major risk factor for equine laminitis and that elevated circulating insulin concentrations can trigger the condition, regardless of whether the animal is insulin-resistant or not [2, 3]. Insulin-dysregulated horses and ponies can have tissue resistance to the effects of insulin resulting in persistent hyperinsulinemia, but alternatively can simply experience an abnormally large post-prandial insulin response to carbohydrate-rich meals [4]. Strategies that attenuate this insulin response would be of considerable therapeutic value in reducing laminitis risk.

The exaggerated post-prandial insulin response exhibited by insulin-dysregulated animals is related to a hyper-responsiveness to glucose and other sugars (non-structural carbohydrates [NSC]) in the diet [4, 5]. Ingested sugars are sensed by a hetero-dimer of two G-protein coupled receptor subunits known as T1R2/3 (taste type 1 receptors 2 and 3), located on the tongue [6]. These receptors are also located on epithelial and entero-endocrine K and L cells in the upper gastrointestinal tract in many species, including horses [7–9]. Activation of these receptors in the small intestine facilitates the absorption of glucose into the bloodstream, which stimulates insulin secretion [10]. Pancreatic insulin secretion occurs primarily in response to glucose, but it is also augmented by incretin hormones, glucagon-like peptide-1 (GLP-1) and glucose-dependent insulinotropic polypeptide (GIP), that are released in response to ingested NSC [11–13]. Incretin release is a key factor in the pathogenesis of metabolic diseases of humans and other animals [4, 14, 15]. Further, T1R2/3 have been directly implicated in the genesis of metabolic dysfunction [16].

The inhibition of sweet taste perception has been investigated for both nutritional and therapeutic purposes [17, 18]. Lactisole ((±)-2-(p-methoxyphenoxy) propionic acid), a T1R3 antagonist, is effective at reducing sweet taste sensation in humans, primates and mice, but not rats [19–21]. By comparison, extracts of *Gymnema sylvestre* contain multiple active taste compounds, including gymnemic acid and gurmarin, which are naturally-occurring T1R2/3 antagonists that effectively inhibit sweet taste, intestinal glucose uptake and incretin release [22–24]. Gymnemic acids show no inhibitory effect on taste in mice and rats, whereas in old world monkeys and humans sweet taste was affected [25–27]. Conversely, gurmarin inhibits sweet perception in rats, mice and gerbils, but not in humans [17, 28, 29]. The capacity of these compounds to inhibit glucose uptake in horses has not been investigated, and their activity on the equine sweet taste receptor is unknown. The aims of the current study were to 1) determine the efficacy of lactisole and *G sylvestre* in reducing glucose uptake by equine small intestine *in vitro* and 2) determine whether lactisole and *G sylvestre* can reduce post-prandial insulin secretion following a carbohydrate-based meal in ponies *in vivo*.

## Materials and methods

### Study *in vitro*

#### Samples

Approval for tissue use was granted by the Animal Ethics Committee of Queensland University of Technology (1400000039). Equine duodenal tissue samples (10 cm length collected 30 cm distal to the pylorus) were obtained immediately upon euthanasia of healthy horses (*Equus caballus*, *n* = 4, 5–15 years old) at a local abattoir (Meramist Pty Ltd, Caboolture, Australia, AUS-MEAT accredited). They were rinsed in cold, sterile saline (0.9%; Baxter Healthcare; Old Toongabbie, NSW, Australia), blotted and placed in oxygenated Tyrode’s cell buffer (TCB: 135 mM NaCl, 5 mM KCl, 1 mM MgCl2, 1.8 mM CaCl_2_, 20 mM Hepes and 0.05% (W/V) BSA at pH 7.4) on ice for transportation (10 min) to the laboratory, where the serosal layer was dissected away and the remaining mucosal tissue sectioned into explants (~500 mg) [30]. The explants were then immediately pre-incubated for 10 min at 30°C in TCB with oxygenation (95% oxygen and 5% carbon dioxide).

#### Inhibition of glucose uptake

The explants were placed individually into glass tubes containing TCB (10 mL) and received one of three treatments: 1) no drug added (positive control); 2) 5 mM lactisole (Sigma Aldrich, NSW, Australia) or 3) 0.3 mg/mL *G sylvestre* (Nature’s Answer, NY, USA). These doses were selected based on existing literature [20, 31]. Each reaction was performed in triplicate. After equilibration (10 min), 1 mL of 2-deoxyglucose (100 mM) was added to each tube. One replicate of tubes that did not contain 2-deoxyglucose were also included as a negative control. Samples were incubated for 10 min at 30°C with oxygenation. Following the incubation, the explants were removed from the solution, blotted and then washed twice with phosphate buffered saline (1%) before being rapidly frozen in liquid nitrogen and stored at -80°C until further analysis.

#### Gas chromatography-mass spectrometry (GC-MS)

Each explant was defrosted on ice in water (0.5 mL; GC-MS grade), homogenised (OMNI International, GA, USA) and the protein concentration quantified using a bicinchoninic acid assay (CV = 3%; Thermo Scientific, IL, USA). An equal amount of protein (1 mg) from each sample was then dried down using a vacuum centrifuge (CHRIST® Rotational Vacuum Concentrator 2033 IR/RCV 2–18 CD, Germany) for 120 min at 50°C prior to methanol extraction (0.4 mL; 100%). Deuterated glucose (D-Glucose-6, 6-d2; Sigma Aldrich, NSW, Australia) was added to each sample (100 parts per billion) as an internal standard (IS). Samples were then centrifuged (10 min; 16400 x *g*; 4°C) and the supernatant retained. Each sample was then dried down again by vacuum centrifuge (45 min; 50°C) in preparation for derivatisation. Meox solution (30 mg/mL) was prepared by mixing methoxyamine (Sigma Aldrich, NSW, Australia) with pyridine (Sigma Aldrich, NSW, Australia) in a glass auto-sampler vial and incubating for 5 min at 50°C. The Meox solution (20 μL) was then added to each sample and incubated for 2 h at 37°C in a thermomixer (Eppendorf AG, Hamburg, Germany) at 500 rpm prior to the addition of 40 μL of N, o-Bis (Trimethylsilyl) trifluoroacetamide in 1% trimethylchlorosilane (Thermo Scientific, IL, USA) and further incubation (40 min; 37°C).

The GC-MS methods were initially validated using six sugars (ribose, xylose, lyxose, glucose, galactose and mannose) and the ion selection optimised with product ion scans. Samples (1 μL) were injected using an auto-injector (Shimadzu AOC-20i, NSW, Australia) into a chromatograph (Shimadzu GC-MS TQ 8040) equipped with a DB-5MS UI capillary column (30 m x 0.25 mm ID, 0.25 μm film thickness). The injector temperature was set at 300°C and the carrier gas was helium with a flow rate of 1 mL/min and a split ratio of 20. The temperature of the column was initially held for 2 min at 170°C, and then elevated by 50°C/min up to 270°C, followed by a 5°C/min increase up to 290°C and then a 30°C/min increase before reaching the final temperature of 320°C, which was held for 1 min. The GC output was then introduced to the TQ 8040 triple quadrupole MS. The MS analysis was carried out in electronic ionisation mode where the data were recorded in full scan or Multiple Reaction Monitoring (MRM) mode (S1 Table). The full scan mass range was 50–600 m/z. The ion source and interface temperature was 280°C with a solvent cut of 2.8 min.

### Study *in vivo*

#### Subjects

Approval for animal use was granted by the University of Queensland Animal Ethics Committee (SVS/QUT/109/13/QUT). Eight, mixed-breed ponies (14 ± 2.8 years, 3 female, 5 male) were selected for the project following a power calculation (mu(0) 50, mu(1) 20, sigma 30, 1-sided test, alpha 0.05, power 0.8) from a herd of 12 ponies, based on behaviour and their metabolic status. The ponies were examined by a veterinary surgeon and found to be in good health, except for their body condition score (BCS), which was above average in most ponies. Subjective analyses of body condition (BCS (1-9/9) [32] and cresty neck score (CNS; 1-5/5) [33]) were performed by two experienced operators, who were blinded to the other’s assessment, and the median BCS of the ponies (7.5 [6–8]) reflected that only one of the ponies was considered to be an ideal BCS (4/9), with the remaining 7 ponies overweight (BCS ≥ 6) at a mean bodyweight (BW) of 186 ± 29 kg. The CNS showed high variability (0 to 4/5; median 2.5 [0.25–3]). All of the ponies were within the defined reference ranges for routine biochemical and haematological parameters. None of the ponies were lame, nor did they develop lameness during the study.

Each pony also underwent an oral glucose test (OGT) to examine their basal and post-prandial serum insulin and blood glucose concentrations (samples taken at 0, 90 and 180 min) to ensure that a sufficient response to oral NSC was present prior to undertaking the study. The OGT demonstrated that all ponies exhibited marked post-prandial responses to oral D-glucose, with insulin concentrations increasing from 3.01 ± 0.7 μIU/mL at time 0, to 41.8 ± 7.65 μIU/mL at 90 min and 32.6 ± 7.3 μIU/mL at 180 min (*P* < 0.01). Thus, all ponies were deemed suitable for inclusion in the study. Throughout the dose-response studies the two compounds were well-tolerated with no adverse events associated with their use.

#### Study design

The efficacy of lactisole (Sigma Aldrich, NSW, Australia) and *G sylvestre* (Nature’s Sunshine, NSW, Australia) in reducing the insulin response to oral D-glucose was measured during two separate dose-response studies performed with a 2 week wash-out period between studies, which was deemed generous based on the short duration of effect of both compounds [20, 34]. Using data from previous human trials as a guide [35, 36], lactisole was administered in parts per million (ppm) at 0, 50, 150 and 500 ppm of the NSC content of the meal provided; whereas *G sylvestre* was administered at 0, 3, 10 and 20 mg/kg BW. The compounds were administered to each pony with the morning feed at 0800 over four consecutive days, with dose levels allocated using a Latin square design and ponies randomly allocated to a starting dose. The diet consisted of 0.3% BW lucerne chaff, plus 200 g wheat bran that has been soaked in a 200 mL solution of D-glucose in water, at a rate of 0.75 mg/kg BW. Feed refusals were collected and weighed 2 hours after feeding. An evening meal of 1.7% BW lucerne hay was provided at 1700, with a vitamin and mineral supplement. Feed refusals were removed at 2100.

Extended-use, intravenous, 14 gauge catheters (Mila International, KY, USA) were placed in a jugular vein (a different vein was used for each study) of each pony under local anaesthesia (2% lignocaine, Troy, NSW Australia) and sutured in place. Patency was maintained with heparinised saline (1:1000). Treatments were administered in-feed at 0800 following an 11-hour fast. Blood samples (6 mL) were collected at the following time-points: 0, 0.5, 1, 1.5, 2, 3, 4, 5 and 6 hours after the meal was provided. Blood glucose was measured immediately using a handheld glucometer previously validated for use in horses by the investigators [37]. The remaining blood was divided between EDTA and plain tubes to yield plasma and serum, respectively. The EDTA tubes were placed immediately on ice for 10 min, whereas the plain tubes were left at ambient temperature for 60 min, allowing the blood to clot. The tubes were centrifuged (10 min; 1500 x *g*) and the plasma/serum separated and stored for later insulin, GIP and active GLP-1 analyses using validated ELISAs (Insulin; Mercodia, Uppsala, Sweden and GIP/aGLP-1; Merck Millipore, MA, USA) [4, 38]. Absorbance was measured at 450 nm using a plate reader (Versamax, CA, USA). It is important to note that the Mercodia insulin assay yields insulin concentrations that are precise, but consistently lower than results reported by other insulin assays used commonly for horses [38].

#### Data analyses

The data were distributed normally (Shapiro-Wilk test) and were analyzed parametrically with the exception of the ordinal body condition data which are reported as median [interquartile range]. The GC-MS data were imported into Skyline software v. 3.6 (MacCoss, IN, USA). To determine glucose uptake the area under the curve (AUC) of the 2-deoxyglucose and deuterated 2-deoxyglucose peaks was calculated using a linear regression method. The average AUC for each sample was normalised against a linear standard curve ranging from 0 to 100 parts per million (ppm) of deuterated 2-deoxyglucose. The mean 2-deoxyglucose concentration was compared with and without the inhibitor using a paired t-test. For each treatment *in vivo* the maximum insulin and glucose concentrations (Cmax) and the AUC (hormone vs. time, 0–360 min) were determined for each pony and the mean value for each dose was compared to the corresponding zero dose with Dunnett’s test. The percent inhibition was compared for insulin and glucose within each dose using a paired t-test. Differences in the % inhibition at the 10 mg/kg dose, compared to no dose, was used to determine the presence of the statistical outlier using Grubb’s test. For GIP and aGLP-1 analyses, the AUC of the 0 and 10 mg/kg doses of *G sylvestre* were compared using a paired t-test. Significance was set at *P* < 0.05, and the data are reported as mean ± s.e.m. Data analyses were performed with SigmaPlot v.12.5 (Systat software, CA, USA).

## Results

### Study *in vitro*

**Fig 1:**
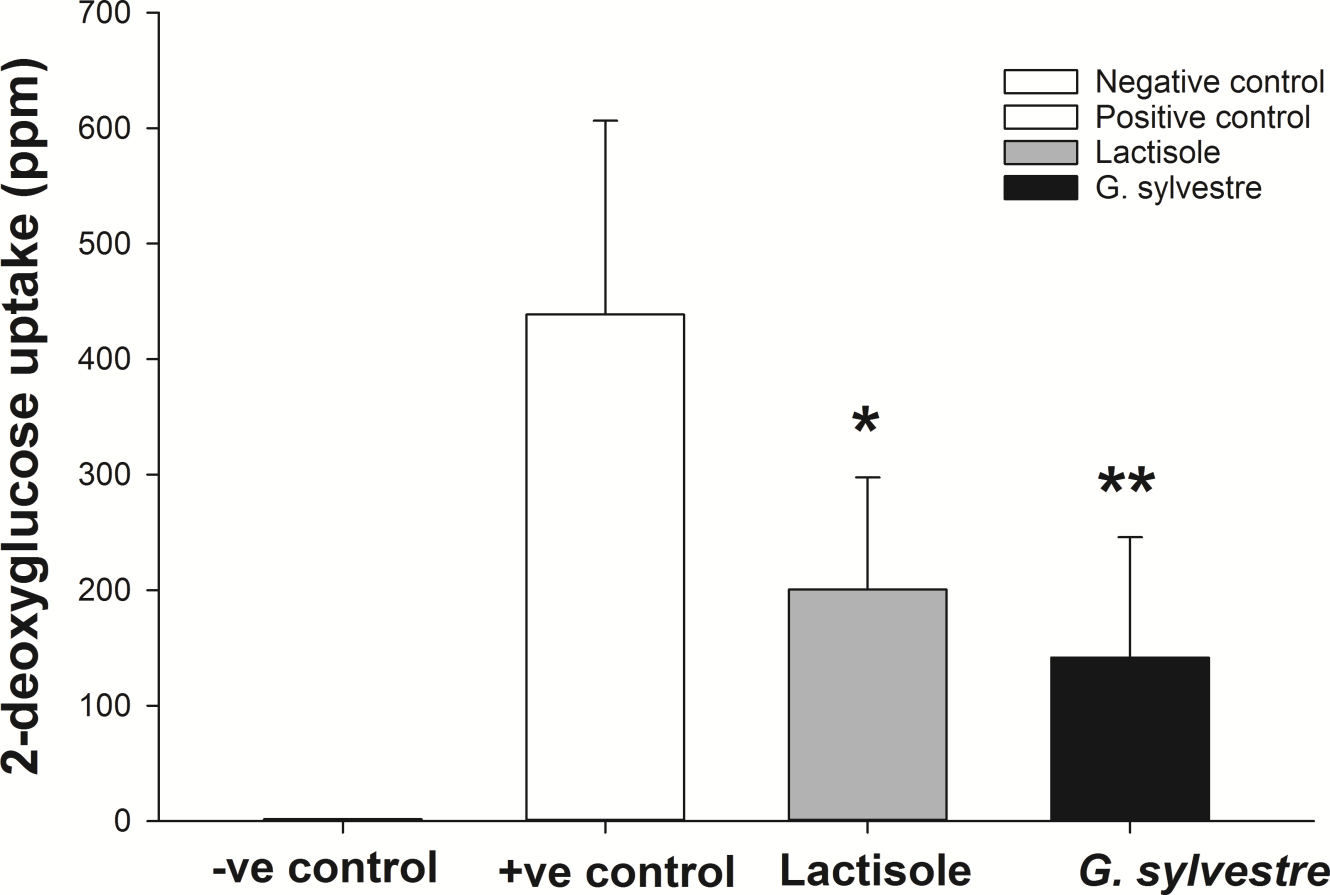
Glucose uptake by the small intestine *in vitro* was inhibited by T1R2/3 antagonists. The mean (± s.e.m.) uptake of 2-deoxyglucose by equine small intestinal tissue (positive control, white bar) following short-term incubation was reduced by 63% (*, *P* = 0.032) by the addition of 5 mM lactisole (grey bar), and by 73% (**, *P* = 0.047) by 0.3 mg/mL *Gymnema sylvestre* (black bar). *P < 0.05.

No uptake of 2-deoxyglucose was detected in the negative control samples, as expected (Fig 1). The glucose uptake by the explants differed markedly between horses, resulting in a large coefficient of variation (76% for the positive control samples). However, compared to each positive control sample, 2-deoxyglucose uptake was reduced markedly by the addition of either lactisole (62.7 ± 8.8%; *P* = 0.032) or *G sylvestre* (73.1 ± 12.1%; *P* = 0.047), as shown in Fig 1.

### Study *in vivo*

There were two incidences of feed refusals associated with the 500 ppm dose of lactisole and the data for these two ponies were removed from the final data set. The addition of lactisole to the D-glucose containing test diet did not reduce the blood glucose response at any dose rate, compared to the meal when no lactisole was added (Table 1). In fact, the glucose Cmax was higher when 50 or 150 ppm of lactisole was added to the feed (*P* < 0.05), and the area under the glucose curve (AUC_glucose_) was also larger at 150 ppm. Similarly, lactisole did not reduce the Cmax or the AUC for insulin (AUC_insulin_) at any dose rate (Table 1). The variation between the ponies in their insulin responses to the meal was considerable (CV = 41% at Cmax).

**Table 1:** Lactisole did not reduce the blood glucose or serum insulin responses to a D-glucose meal in eight ponies at any dose tested.

Dose	0 ppm	50 ppm	150 ppm	500 ppm^§^
**Cmax glucose (mmol/L)**	8.1 ± 0.68	10 ± 0.74*	10 ± 0.73*	9.7 ± 1.15
**AUC_glucose_**	39.9 ± 2.12	45.8 ± 4.01	46.2 ± 3.4*	41 ± 3.53
**Cmax insulin (μIU/mL)**	60.4 ± 9.29	67.2 ± 5.1	75.7 ± 15	58.8 ± 11.9
**AUC_insulin_**	226 ± 48.1	263 ± 29.5	271 ± 70.6	220 ± 57.4

Key

*; *P* < 0.05 compared to 0 ppm

^§^; *n* = 6, AUC; area under the curve, Cmax; maximal response, ppm; parts per million

The addition of *G sylvestre* to the diet also failed to cause any apparent difference in the glucose Cmax, or the AUC_glucose_, at any dose rate (Table 2). However, the compound did appear to cause a dose-dependent decrease in the insulin Cmax, although this decline was not statistically significant at any individual dose rate due to the large degree of variation in response between ponies. The AUC data for insulin demonstrated a similar relationship with the *G sylvestre* dose rate (Table 2).

**Table 2:** *Gymnema sylvestre* did not reduce the blood glucose or serum insulin responses to a D-glucose meal when tested in eight ponies at three dose rates.

Dose (mg/kg BW)	0	3	10	20
**Cmax glucose (mmol/L)**	9.2 ± 0.63	8.3 ± 0.56	8.6 ± 0.97	9 ± 0.74
**AUC_glucose_**	45.2 ± 3.11	41.4 ± 2.41	41.8 ± 3.6	44.1 ± 3.36
**Cmax insulin (μIU/mL)**	82.9 ± 16.3	73.1 ± 17	71.5 ± 15.3	67.3 ± 13.4
**AUC_insulin_**	317 ± 65.5	302 ± 71.1	260 ± 58	252 ± 57.2

Key: AUC; area under the curve, Cmax; maximal response

**Fig 2:**
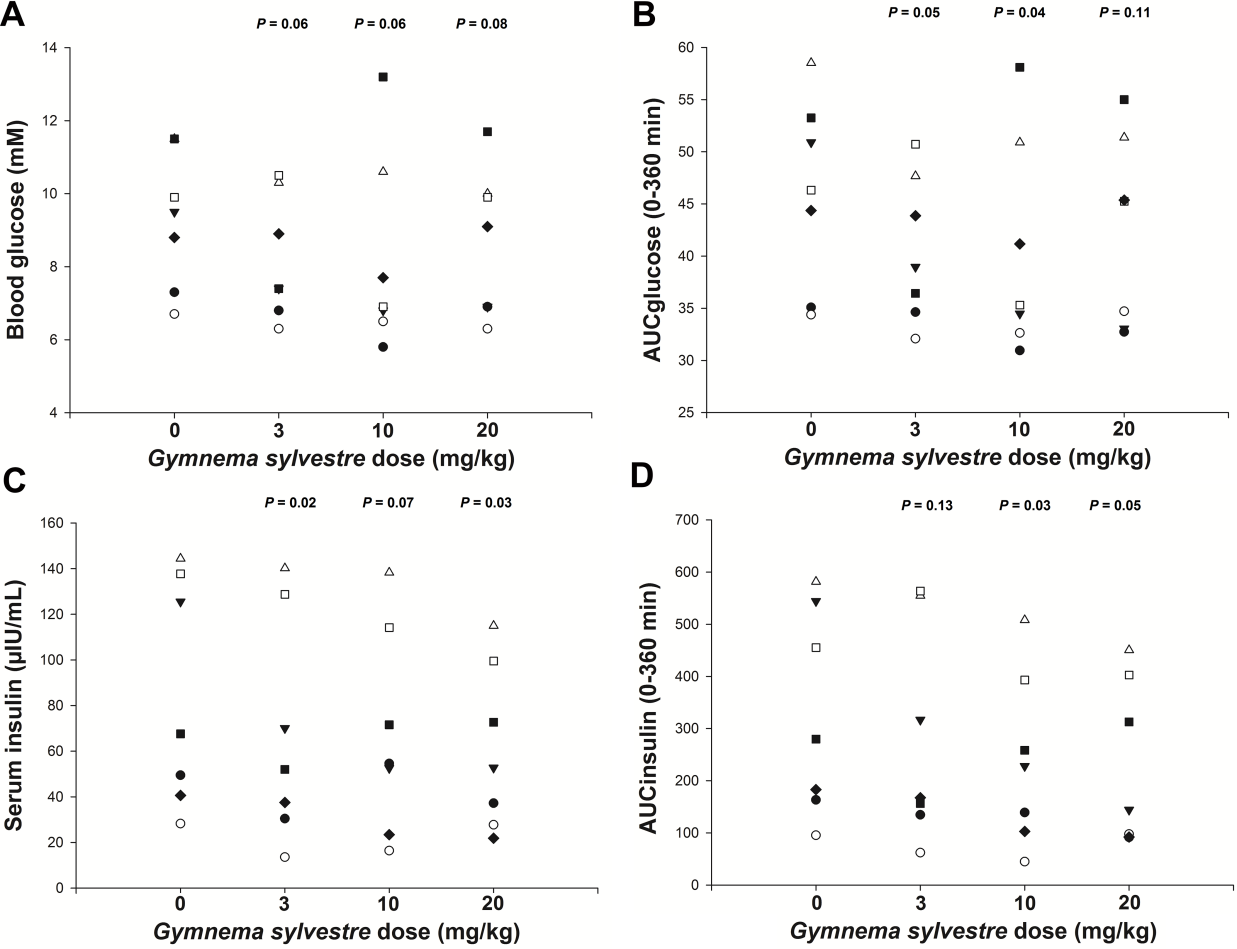
*Gymnema sylvestre* reduced the blood glucose and serum insulin responses in seven ponies. The mean (± s.e.m.) glucose Cmax **(A)** was not reduced when *G sylvestre* was added to a D-glucose meal at any dose rate tested. The mean (± s.e.m.) glucose AUC_0-360 min_ **(B)** decreased with the addition of *G sylvestre* at 3 and 10 mg/kg BW, compared to no added drug. Similarly, the mean (± s.e.m.) insulin Cmax **(C)** decreased when *G sylvestre* was added to a D-glucose meal at 3 and 20 mg/kg, with no decrease at 10 mg/kg, compared to no added drug. Further, the insulin AUC_0-360 min_ **(D)** decreased when 10 and 20 mg/kg BW *G sylvestre* was added. The data for each pony is represented with a different symbol.

On further examination of these data it was evident that one pony did not respond to the addition of the compound at all and this pony was found to be a statistical outlier. When the data from this pony were removed and the data were re-analysed, the glucose Cmax was not reduced (*P* = 0.05–0.1) at any dose rate (Fig 2A), but the AUC_glucose_ was reduced (*P* ≤ 0.05) at 3 and 10 mg /kg BW (Fig 2B). In addition, a reduction in the insulin Cmax occurred with 3 and 20 mg *G sylvestre*/kg BW, while insulin Cmax at 10 mg/kg BW was not reduced (Fig 2C). The AUC_insulin_ was also reduced (*P* ≤ 0.05) at 10 and 20 mg *G sylvestre* /kg BW (Fig 2D).

**Fig 3:**
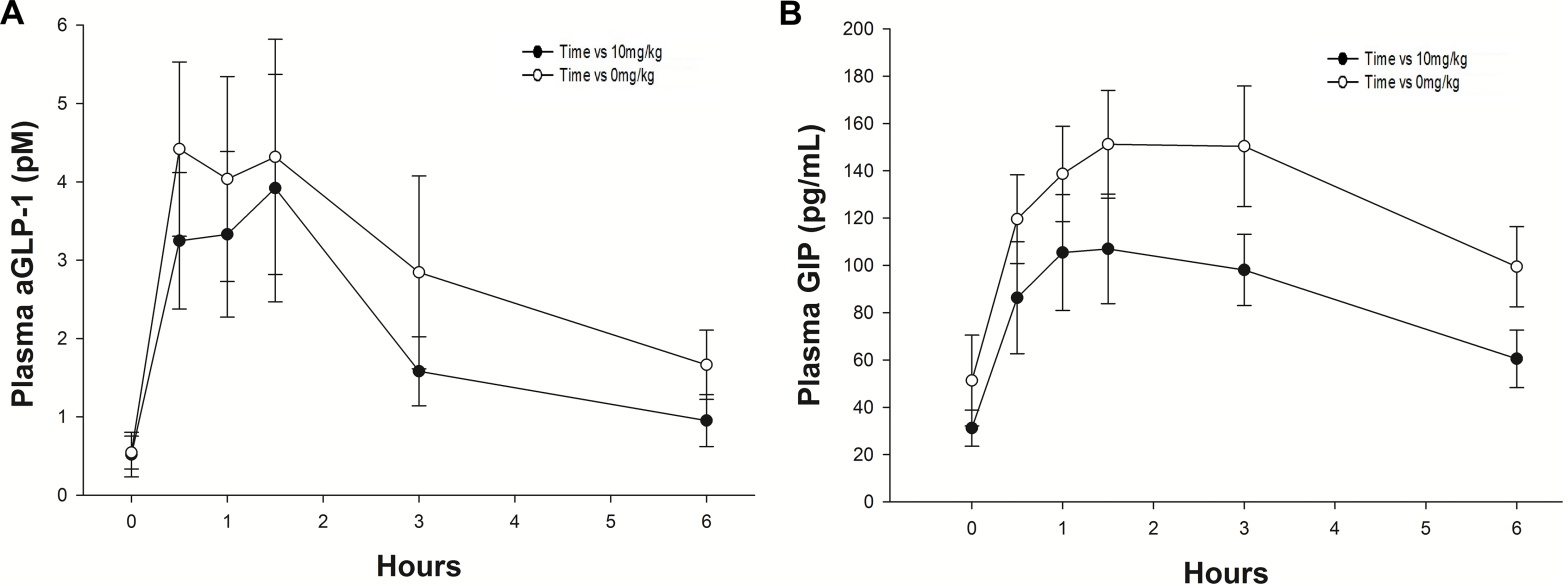
*Gymnema sylvestre* did not appreciably reduce aGLP-1 or GIP secretion in response to a carbohydrate meal in seven ponies. Neither the mean (± s.e.m.) AUC_aGLP-1_ **(A)** nor the mean (± s.e.m.) AUC_GIP_ **(B)** were decreased (*P* = 0.075 and *P* = 0.11, respectively) when *G sylvestre* was added to a D-glucose meal at a dose rate of 10 mg/kg BW.

The percent inhibition of blood glucose and serum insulin (Cmax and AUC) at each dose rate of *G sylvestre* is presented in Table 3. From these data it can be seen that *G sylvestre* had a larger effect on insulin secretion, compared to blood glucose at some doses (n = 7, *P* < 0.05). To further investigate this outcome, plasma incretin (GIP and aGLP-1) responses were determined for the most effective dose overall, which was 10 mg *G sylvestre* /kg BW. However, there was no appreciable reduction in AUC_aGLP-1_ (*P* = 0.075; Fig 3A) or AUC_GIP_ (*P* = 0.11; Fig 3B) with the addition of *G sylvestre*.

**Table 3:** *Gymnema sylvestre* inhibited insulin and glucose responses to a D-glucose meal in seven ponies.

Dose, mg/kg BW	% inhibition glucose Cmax	% inhibition insulin Cmax	% inhibition AUC_glucose_	% inhibition AUC_insulin_
**3**	10.5 ± 5.36	25 ± 7.6	10.5 ± 5.48	18.2 ± 9.18
**10**	12.5 ± 5.95	21.1 ± 10.1	12.0 ± 5.03^§^	29.1 ± 8.16^§^
**20**	6.67 ± 4.04*	24.5 ± 8.66*	7.1 ± 5.09*	26.7 ± 11.5*

Key

*; *P* = 0.03 and

^§^; *P* = 0.04 for comparison between glucose and insulin within a dose, AUC; area under the curve, Cmax; maximal response

## Discussion

Increased awareness of the fact that hyperinsulinemia is a key risk factor for equine laminitis has heightened demand for an effective therapy for insulin dysregulation. Unlike most species, horses rarely develop diabetes mellitus, but instead retain good pancreatic capacity for insulin secretion in response to excessive oral carbohydrates. In an insulin-dysregulated horse hyperinsulinemia can manifest as pulsatile, post-prandial spikes in circulating insulin, or in the development of persistent hyperinsulinemia, which may be associated with insulin resistance [4, 39].

In theory, reducing insulin action in order to reduce laminitis risk could be achieved by limiting insulin secretion, or by inhibiting receptor binding at the target site. However, the pharmacologic options available for treating diabetes mellitus in humans usually have the opposite effect, with the aim of increasing insulin secretion from a failing pancreas, or augmenting insulin action. Thus, novel therapeutic strategies need to be pursued for equine insulin dysregulation and currently, there are no effective pharmacologic options available.

As such, the current study investigated the efficacy of sweet taste receptors on glucose uptake in this species. In doing so we focused downstream of the sweet taste receptor and it is important to note that an assessment of intrinsic activity of the receptor, including sweet taste perception, was not undertaken. The data *in vitro* reported in this study show, for the first time, that both lactisole and *G sylvestre* function as inhibitors of glucose uptake in horses (potentially through blocking the T1R2/3 receptor complex), indicating their potential usefulness in limiting post-prandial insulin secretion. The ability of lactisole and *G sylvestre* to inhibit glucose uptake *in vitro* was consistent and marked, despite the large inter-horse variability in intestinal glucose absorption. The reason for this difference in glucose uptake per mg of tissue between individual animals is unknown. The fact that the replicates from each horse were consistent suggests that the distribution and density of the glucose transporters in the mucosal brush border may differ between individuals. It has been demonstrated in humans and rodents that dietary glucose availability and disease state can regulate the intestinal expression of T1R2/3 [40, 41]. Further, stimulation of the T1R2/3 complex by increased luminal glucose upregulates glucose uptake via the sodium-dependent glucose transporter-1 (SGLT-1) [42]. The upregulation of entero-endocrine cell SGLT-1s leads to increased incretin release and the exacerbation of hyperinsulinemia [42]. The expression of SGLT-1 can also change in response to disease, with increased expression in obese humans [43]. Although SGLT-1 expression during metabolic dysfunction has not been examined in horses, increased post-prandial glucose bioavailability has been reported in insulin-dysregulated ponies, compared to metabolically healthy ones [4]. Thus, the variability in glucose uptake in vitro in the current study may be related to differential expression of T1R2/3 and/or SGLT-1 in the equine small intestine, secondary to variations in dietary history or underlying disease, and this hypothesis requires investigation.

Despite the efficacy of lactisole *in vitro*, the current study did not demonstrate the inhibition of glucose uptake, or subsequently, insulin secretion, by lactisole following a carbohydrate-rich meal in ponies *in vivo*. It is possible that the lactisole doses selected for this study may have been insufficient, but considering the wide dose range used, and the efficacy of these doses in other species, other reasons should be explored. It is possible that the oral bioavailability of lactisole is poor in horses. Despite being a hindgut fermenter, the equine small intestinal tract is not dissimilar to other monogastric species. However, gastroduodenal pH can be labile and the gastrointestinal transit time is sensitive to meal size and frequency due to the relatively small size of the horse’s stomach [44]. Poor oral bioavailability of medications has been reported with reasonable frequency in this species [45–47]. Thus, an investigation of the oral bioavailability of lactisole in horses is warranted, prior to further investigations of efficacy. Further, an investigation of intrinsic activity at the level of the receptor would aid in determining the efficacy of lactisole in this species.

In comparison to lactisole, although *G sylvestre* was not effective in all of the ponies, it appeared to have some effect *in vivo* in the majority of the animals tested. When examined solely in the responders the compound reduced post-prandial blood glucose concentrations by up to 12%. However, the inhibition of 2-deoxyglucose uptake achieved in the *in vitro* study was much greater at 73%. Glucose uptake from the small intestine is affected by numerous factors, as discussed earlier, including intestinal glucose transport capability and gastric motility, and these factors, in addition to first-pass hepatic extraction of glucose, will affect the amount of glucose that appears in the circulation for measurement on a day to day basis. As such, there are numerous factors that could have contributed to the variability in effect between animals and in the overall reduced inhibitory effect of *G sylvestre* detected *in vivo*.

Of primary importance though, is the fact that a different product was used in each of the two phases of the study. A liquid extract of *G sylvestre* was required for the *in vitro* study, but the use of this product was cost-prohibitive *in vivo* due to the large size of the subjects. Thus, a solid oral formulation (dried herb) was used for the pony study and any difference in the quality of the products could have contributed to the variability in response. Further, as discussed above for lactisole, the oral bioavailability of *G sylvestre* may have been sub-optimal [34]. It is unclear why the compound appeared to have failed completely in one animal, at all of the doses tested, but we suggest that any or all of the above factors could have resulted in this lack of efficacy.

At all of the doses of *G sylvestre* tested, the reduction in serum insulin concentrations (~25%) was approximately double the inhibition achieved for blood glucose. The finding that the percent inhibition of insulin was larger than the inhibition of glucose within the same animals is not physiologically surprising. Plasma glucose concentrations in a normal animal are maintained in a narrow physiological range and do not approach zero at any time, but plasma insulin can be expected to approach zero in a fasted animal, which demonstrates that within the range of normal physiology, differences in insulin can be greater than changes in glucose without any additional processes being invoked [48]. However, with the knowledge that T1R2/3 receptors are present directly on entero-endocrine cells we elected to further investigate the reduction in insulin by measuring incretin hormone concentrations [8].

The presence of T1R2/3 receptors on K and L cells has been demonstrated to trigger the release of GIP and GLP-1, respectively, which, in turn, enhances pancreatic insulin secretion [10, 13]. Accordingly, antagonism of T1R2/3 signalling on these cells should further contribute to the inhibition of insulin secretion, above and beyond what was achieved through the direct reduction in blood glucose concentration. However, the finding that a notable reduction in the incretin hormones did not occur with *G sylvestre* administration, failed to support this supposition. Previous studies on incretin release in ponies have reported that the incretin effect in less substantial in horses, than other species such as humans [4]. Thus, the lack of effect of the T1R2/3 inhibitors on aGLP-1 and GIP secretion in this study may be related to the subdued equine incretin effect [4]. However, we must acknowledge that the sample size in the current study was small, and although power calculations were undertaken the large variability in insulin responses did affect the data analyses in this study. Further, studies that specifically show the efficacy of *G*. *sylvestre* at the sweet taste receptor in this species would clarify these data. As such, further studies are required to more fully determine if there is a potential role for the incretins in the mechanism of action of *G*. *sylvestre*.

Despite the fact that these combined data support that T1R2/3 inhibitors can function to inhibit insulin secretion via two separate pathways in horses, the relatively small reduction in post-prandial insulin Cmax achieved (~ 20 μIU/mL) by *G sylvestre* may not be relevant clinically, given that the response was not strongly dose-dependent. Ponies with insulin dysregulation can produce post-prandial serum insulin concentrations well over 100 μIU/mL. Thus, presumably a larger reduction in insulin Cmax would be required than was achieved, even at the largest dose tested, to be effective at reducing laminitis risk. However, these preliminary data are supportive of the need for further investigation into the potential dose-response relationship of *G sylvestre*, and its potential as a treatment for insulin dysregulation in horses. Initially, pharmacokinetic studies need to be undertaken in combination with data modelling to enable dose selection prior to further studies *in vivo*. In addition, long term safety studies have not been undertaken. Studies that have administered *G sylvestre* to rats for up to 4 weeks at a much higher dose rate (120 mg/kg BW) reported no adverse drug-associated effects [49]. This suggests that the compound may be well-tolerated in the longer term, however species differences in drug metabolism mean that safety studies in the target species are essential.

In conclusion, the current study has provided foundation data for a novel therapeutic strategy for equine insulin dysregulation. These preliminary data could be used as a baseline for the design and planning of further research that will investigate the safety, efficacy and feasibility of this approach to managing equine insulin dysregulation.

## Supporting information

S1 Table Gas chromatography-mass spectrometry conditions used to quantify 2-deoxyglucose. (DOCX)
