# Supplementary material for: Sweet taste receptor inhibitors: Potential treatment for equine insulin dysregulation
Source: PLoS One. 2018 Jun 29;13(6):e0200070. doi: 10.1371/journal.pone.0200070 (PMC6025858; doi:10.1371/journal.pone.0200070)
Supplement: S1 Table — (DOCX) [file pone.0200070.s001.docx]

**S1 Table: Gas chromatography-mass spectrometry conditions used to quantify 2-deoxyglucose**

| Scan Mode | Parent Ion | Product ion | Collision energy |
| --- | --- | --- | --- |
| Multiple Reaction Monitoring | 217 | 129 (Quantifier) | 9 |
| Multiple Reaction Monitoring | 204 | 189 (Confirmation) | 8 |
